# Supplementary figures and images for: Diversity and Co-Occurrence Pattern Analysis of Cecal and Jejunal Microbiota in Two Rabbit Breeds
Source: Animals (Basel). 2023 Jul 13;13(14):2294. doi: 10.3390/ani13142294 (PMC10376057; doi:10.3390/ani13142294)

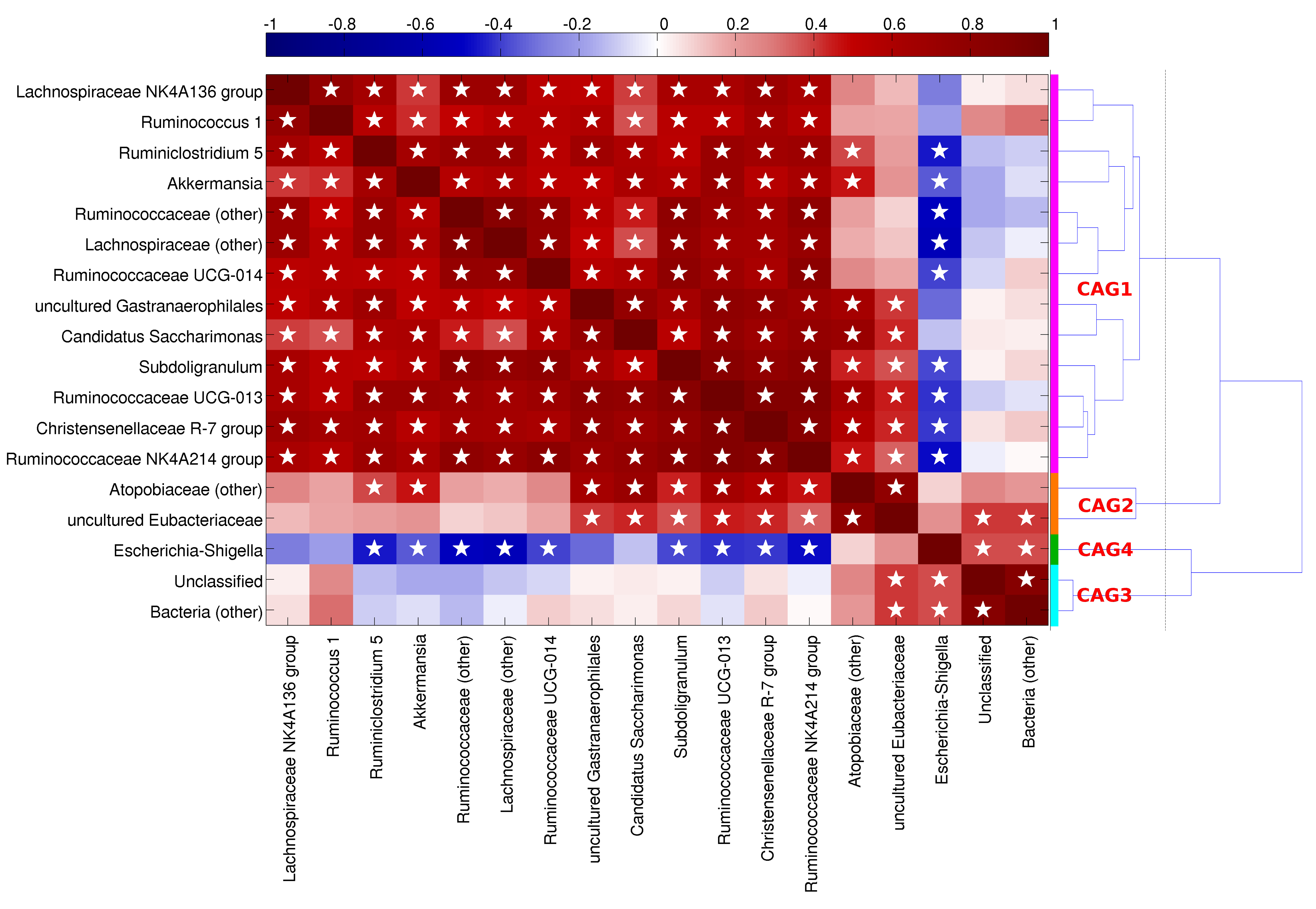

Supplement: Supplementary file 1 [file animals-13-02294-s001.zip › Supplementary Files, Figure S1.tiff]
